# ISL1-based LIM complexes control *Slit2* transcription in developing cranial motor neurons

**DOI:** 10.1038/srep36491

**Published:** 2016-11-07

**Authors:** Kyung-Tai Kim, Namhee Kim, Hwan-Ki Kim, Hojae Lee, Hannah N. Gruner, Peter Gergics, Chungoo Park, Grant S. Mastick, Hae-Chul Park, Mi-Ryoung Song

**Affiliations:** 1 School of Life Sciences, Gwangju Institute of Science and Technology, Oryong-dong, Buk-gu, Gwangju 500-712, Republic of Korea; 2 Graduate School of Medicine, Korea University, Ansan 425-707, Korea; 3 Department of Biology, University of Nevada, Reno, NV 89557, USA; 4 Department of Human Genetics, University of Michigan, 1241 Catherine St, Ann Arbor, Michigan, MI-48109, USA; 5 School of Biological Sciences and Technology, Chonnam National University, 77 Yongbong-ro, Buk-gu, Gwangju 500-757, Republic of Korea

## Abstract

LIM-homeodomain (HD) transcription factors form a multimeric complex and assign neuronal subtype identities, as demonstrated by the hexameric ISL1-LHX3 complex which gives rise to somatic motor (SM) neurons. However, the roles of combinatorial LIM code in motor neuron diversification and their subsequent differentiation is much less well understood. In the present study, we demonstrate that the ISL1 controls postmitotic cranial branchiomotor (BM) neurons including the positioning of the cell bodies and peripheral axon pathfinding. Unlike SM neurons, which transform into interneurons, BM neurons are normal in number and in marker expression in *Isl1* mutant mice. Nevertheless, the movement of trigeminal and facial BM somata is stalled, and their peripheral axons are fewer or misrouted, with ectopic branches. Among genes whose expression level changes in previous ChIP-seq and microarray analyses in *Isl1*-deficient cell lines, we found that *Slit2* transcript was almost absent from BM neurons of *Isl1* mutants. Both ISL1-LHX3 and ISL1-LHX4 bound to the *Slit2* enhancer and drove endogenous *Slit2* expression in SM and BM neurons. Our findings suggest that combinations of ISL1 and LHX factors establish cell-type specificity and functional diversity in terms of motor neuron identities and/or axon development.

Motor neurons (MNs) transmit signals from the CNS to peripheral muscles to control voluntary and involuntary movements. For instance, cranial motor neurons, which control head and neck movements comprise three subtypes based on their functions and origins: branchiomotor, visceral motor (VM) and somatic motor neurons. BM neurons control the movement of tongue and jaw and facial expression, and VM neurons regulate involuntary movement as part of the autonomic nervous system. SM neurons innervate skeletal muscles and control voluntary movements. The origins and major transcription programs of BM/VM neurons differ from those of SM neurons such as PHOX2 and ISL1 factors for BM/VM neurons and LHX3 and ISL1 for SM neurons^1^,^2^,^3^. However, it is unclear whether the signals for targeting the axons of individual cranial motor neurons to distinct muscle targets are the same or different^4^,^5^.

ISLET1 (ISL1) is a member of the LIM-HD transcription factor family present in all MNs. Its role in acquisition of motor neuron identity in the spinal cord is well-established^6^,^7^,^8^,^9^,^10^. When ISL1 expression is reduced, SM neurons transdifferentiate into V2a interneuron-like cells in the spinal cord^2^,^8^. ISL1 is also expressed in postmitotic MNs, raising the possibility that it plays additional roles. Indeed, several lines of evidence suggested that it may control MN axon pathfinding. In zebrafish, peripheral projections of RB primary sensory neurons and trigeminal neurons are affected when *isl1* or its paralogue *isl2* is mutated^7^,^9^,^11^. Axon pathfinding and neurotransmitter identity are compromised in some neurons in *isl1* null flies^12^. In mice, when the ISL1 level is reduced, peripheral projections of retinal axons as well as motor and sensory neurons are disrupted, all of which support the potential roles of ISL1 in axon navigation^13^,^14^.

LIM-HD transcription factors build multimeric complex via interaction with LBD1 as demonstrated by previous biochemical, structural and genetic studies^2^,^15^,^16^,^17^,^18^. Unlike other LIM-HD family members, ISL1 appears to act together with other LHX factors in the CNS such as spinal cord motor neurons and striatal interneurons^2^,^19^. However, it is uncertain whether a similar combinatorial LIM-code is employed in most neurons in general. For instance, BM neurons in the hindbrain also express ISL1 but no LHX factors that interact with ISL1 are known. Furthermore, diverse combinatorial LIM-codes may control multiple biological functions in the same or different cells.

To investigate the postmitotic roles of ISL1-based LIM-complex in axon guidance, we focused on cranial BM neurons, which retain their identity and project axons to the periphery in the absence of ISL1^8^. We found that, in *Isl1* compound mutant mice, many subpopulations of cranial motor axons were defasciculated or misrouted. Notably, misprojection of BM axons accompanied arrest of the movement of BM somata, indicating that both peripheral and central mechanisms were affected in the absence of ISL1. Analyzing previous Chip-seq and microarray experiments and *in situ* hybridization of candidate genes revealed that *Slit2* transcription was controlled by ISL1, with the help of LHX4, and that *Slit2* mRNA levels were downregulated in *Isl1*-deficient BM neurons. We therefore suggest that *Slit2* is a downstream target of ISL1 in cranial motor neurons and ISL1 controls axon pathfinding in cranial motor neurons.

## Results

### Specification of cranial motor neurons in *Isl1* compound mutant mice

**Figure 1:**
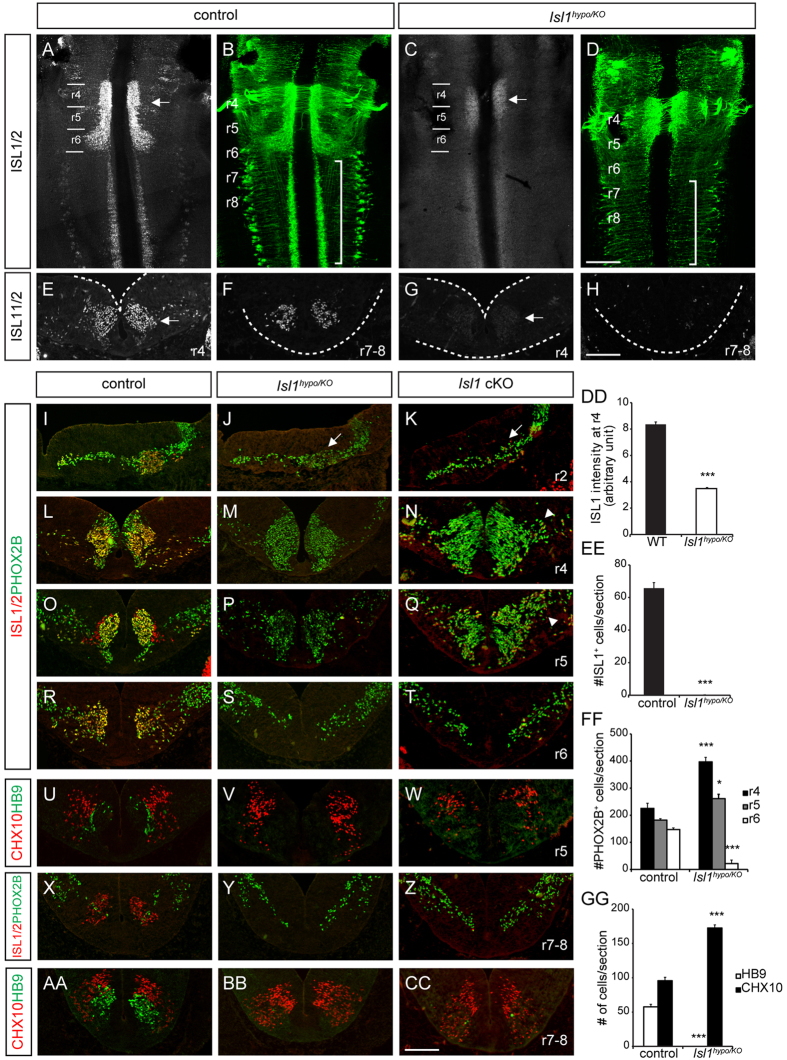
Specification of cranial motor neurons in *Isl1*^*hypo/KO*^ mice. (**A,C,E–H**) Immunostaining of ISL1 in flat-mounted preparations and transverse sections of E11.5 hindbrains. ISL1 immunoreactivity is reduced in r4 (compare arrows in **A,E**,**C,G**) and absent in r7–8 (**H**). (**B,D**) Flat-mounted preparation of E11.5 *ISL*^*MN*^*: GFP-F* hindbrains. SM neurons are missing from the caudal brain in the *Isl1* compound mutant mice (compare brackets in **B,D**). (**I–CC**,**FF,GG**) Immunostaining and quantification of the BMN marker PHOX2B, the SMN marker HB9, V2a interneuron marker CHX10 and ISL1/2 in the E11.5 transverse hindbrain sections. In both *Isl1* compound and cKO hindbrains, FBM neurons stall at r5 and Hb9^+^ SM neurons are absent from caudal hindbrains, in which more CHX10^+^ V2a interneurons arise. Note that the r2 trigeminal nuclei are smaller (arrows, **J,K**) and some r4 FBM neurons migrate laterally (arrowheads, **N,Q**) in the *Isl1* mutants (**FF**: n = 6; (**GG**) n = 6; number of sections). (**DD,EE**) ISL1 immunofluorescence intensity in r4 and the number of ISL1^+^ cells in r7–8. n = 45 (**DD**), n = 18 (**EE**). Error bars represent s.e.m. *p < 0.05 compared with control, ***p < 0.001 compared with control, unpaired Student’s t-test in (**DD,EE,GG**), Mann-Whitney rank sum test in (**FF**). Scale bars: in (**D**), 250 μm for (**A–D**) in (**H**), 100 μm for (**E–H**); in (**CC**) 100 μm for (**I-CC)**.

*Isl1* null mutants do not survive beyond E9.5 due to cardiovascular defects, which make it impossible to investigate their neural development^10^. We instead used *Isl1* compound mice (*Isl1*^*hypo/KO*^) carrying one *Isl1* hypomorphic allele and one *Isl1* null allele^8^,^10^,^14^. A significant reduction in ISL1 immunoreactivity in the spinal cord was observed in similar *Isl1* mutant mice with an *Isl1* hypo allele and an *Isl1*^*nLacZ*^ knock-in allele^14^. Our *Isl1* compound mice survived until E11.75, which allowed to examine the roles of ISL1 in motor neurons. We also used *Isl1* conditional knockout (cKO) mice with a CNS-specific *Nestin-Cre* that efficiently removed ISL1 protein in the hindbrain^8^. In wild-type E11.5 hindbrain flat-mount tissues and transverse sections, ISL1 is present in all cranial motor neurons including the facial branchiomotor (FBM) neurons, which migrate from r4 to r6 (Fig. 1A,E). Trigeminal (V) in r2 and FBM (VII) neurons in r4 to r6 co-expressed PHOX2B and ISL1 (Fig. 1I,L,O,R)^3^,^4^. In the *Isl1* compound mutant mice, ISL1 immunoreactivity was reduced by 40% in r4 FBMs and undetectable in caudal hindbrains (r7–8) (Fig. 1C,G,H,DD,EE). To trace cranial motor neurons when the ISL1 level was low, we crossed *Isl1* compound mutant mice with *ISL*^*MN*^*: GFP-F* transgenic mice in which all motor neurons are GFP-labeled^20^. Flat-mounted hindbrains of E11.5 littermate control mice showed all cranial motor somata and axons labeled by GFP (Fig. 1B). In flat-mounted hindbrains of the *Isl1* compound mutants, FBM neurons were present in r4 and r5 and a few SM somata were in the caudal hindbrain (Fig. 1D). We also labeled *Isl1* mutant neurons with a *delta-Isl1* riboprobe designed to detect partial non-functional transcripts that overlap with *Tbx20* transcripts, which would indicate that ISL1-low cells survive and express BM neuronal markers (Supplementary Figure S1A–R). Mutant BM neurons labeled with PHOX2B and ISL1/2 were comparable in number with those in their littermate control in the r2 trigeminal nerve, while greater numbers of r4 FBM neurons were dispersed laterally in the *Isl1* compound and cKO mutants (Fig. 1I–N). Overall, the generation and initial specification of the trigeminal and FBM neurons appeared relatively normal in *Isl1* mutant mice.

Next we tested whether BM nuclei were correctly positioned after cell body migration: trigeminal somata move from medial to lateral at r2, and FBM somata migrate tangentially from r4 to r6^21^. Trigeminal neurons migrated normally but their nuclei were smaller in *Isl1* compound mutant mice and, to a greater degree, in *Isl1* cKO mice, indicating that ISL1 is required for lateral migration of trigeminal neurons (Fig. 1I–K). FBM neurons in transverse sections of r4 to r6 adopt characteristic shapes in a medial position in r4 and r5, and a lateral position in r6 (Fig. 1L,O,R). In *Isl1* compound and cKO mice, most FBM neurons remained in r4 and r5 and failed to arrive at r6 (r4; control, 225.3 ± 19.2 cells; *Isl1*^*hypo/KO*^, 397.0 ± 16.5 cells, *p* < 0.001), (r5; control, 182.3 ± 5.1 cells; *Isl1*^*hypo/KO*^, 261.3 ± 16.2 cells, *p* = 0.029), (r6; control, 147.3 ± 5.9 cells; *Isl1*^*hypo/KO*^, 21.8 ± 13.1 cells, *p* < 0.001) (Fig. 1M,N,P,Q,S,T,FF). In addition, r4 and r5 FBM neurons tended to spread laterally in *Isl1* compound mutant mice, and more so in *Isl1* cKO mice (Fig. 1N,Q). Together these observations indicate that FBM neurons arise normally when the ISL1 level is low, but their migration is disrupted.

We examined whether the SM neurons in r5 and the caudal hindbrain are intact in *Isl1* compound mutant mice, despite the absence of ISL1immunoreactivity (Fig. 1EE). Previously, elimination of ISL1 in the spinal cord was found to result in an increase of V2a interneurons at the expense of SM neurons^8^. Similarly, we observed that HB9-expressing motor neurons disappeared (control, 57.5 ± 3.8 cells; *Isl1*^*hypo/KO*^, 0.0 ± 0.0 cells, *p* < 0.001) and CHX10^+^ V2a interneurons appeared (control, 95.6 ± 5.0 cells; *Isl1*^*hypo/KO*^, 172.5 ± 4.5 cells, *p* < 0.001) in r5 and r7–8 (Fig. 1V,W,BB,CC,GG). Thus, the production of SM neurons is disrupted in *Isl1* compound mutant mice.

### BM axons are defective in *Isl1* compound mutant mice

**Figure 2:**
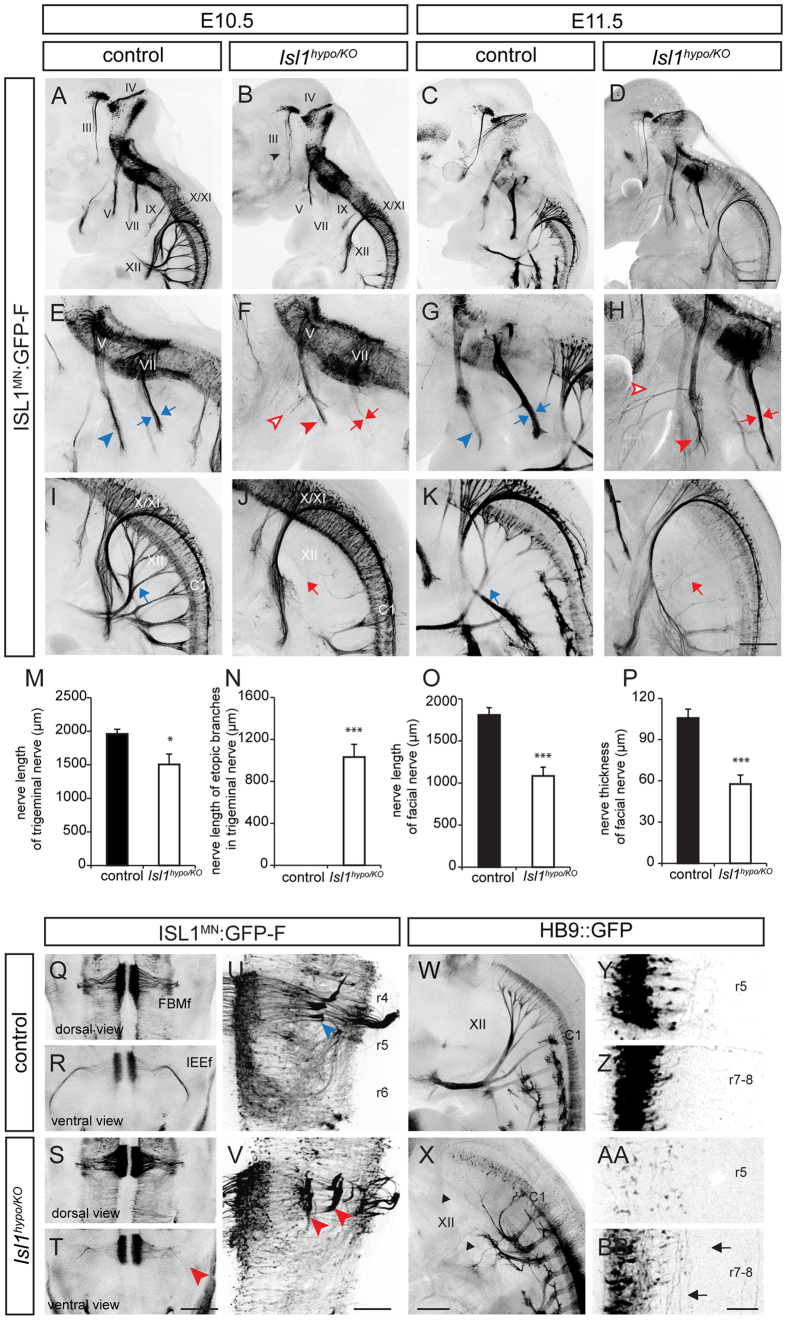
Axon defects of cranial MNs in *Isl1*^*hypo/KO*^ mice. (**A–D**) Wholemount GFP immunostaining of E10.5 and E11.5 *ISL*^*MN*^*: GFP-F* reporter embryos. Oculomotor axons of *Isl1*^*hypo/KO*^ mice are defasciculated at E10.5 (arrowhead, **B**). (**E–L**) Magnified views of images in (**A–D**). The trigeminal mandibular (**V**) nerves of *Isl1*^*hypo/KO*^ mice are defasciculated (arrowhead in **E,G** vs arrowhead in **F,H**) and develop ectopic branches (open arrowheads, **F,H**). The facial nerve (VII) are fewer (arrows in **E,G** vs arrows in **F,H**) and the hypoglossal nerve (XII) is absent (arrow in **I,K**, arrow in **J,L**) (E10.5: n ≥ 3; E11.5: n ≥ 7; number of embryos). (**M–P**) Quantification of nerve length and thickness of axons. Error bars represent s.e.m. *p < 0.05, ***p < 0.001, unpaired Student’s t-test. (**Q–T**) Open-book flat-mount preparation of E11.5 hindbrains. IEE projections are short and disorganized in *Isl1*^*hypo/KO*^ mice (arrowhead, **T**) (control: n = 5; *Isl1*^*hypo/KO*^ mice: n = 3; number of embryos). (**U,V**) Flat-mounted preparation of E11.5 hindbrains. Migration of FBM neurons is arrested in r5 and their exit points are disrupted (arrowhead in **U** vs arrowheads in **V**) (control: n = 4; *Isl1*^*hypo/KO*^ mice: n = 3; number of embryos). (**W,X**) Whole-mount views of E11.5 *Hb9::GFP* mice. Axons of hypoglossal (XII) neurons and spinal cord MNs in *Isl1*^*hypo/KO*^ mice are severely reduced or missing (arrowheads) (control: n = 4; *Isl1*^*hypo/KO*^ mice: n = 3; number of embryos). (**Y-BB**) Flat-mount views of E11.5 *Hb9::GFP* hindbrains. The abducens neurons in r5 and SM somata are reduced and the interneuron-like longitudinal projections were found in the *Isl1*^*hypo/KO*^ mice (arrows, **BB**) (control: n = 5; *Isl1*^*hypo/KO*^ mice: n = 2; number of embryos). III, oculomotor; (**V**) trigeminal mandibular; VII, facial nerve; IX, glossopharyngeal; X, vagal; XI, spinal accessory; XII, hypoglossal nerve; FBMf, facial branchio motor neuron fibers; IEEf, inner ear efferent fibers. Scale bars: in (**D**), 500 μm for (**A–D**) in (**L**), 250 μm for (**E–L**) in (**T**), 500 μm for (**Q–T**) in (**V**), 200 μm for (**U,V**) in (**X**), 250 μm for (**W,X**) in (**BB**), 200 μm for (**Y-BB**).

To trace the axonal projections of BM/VM neurons, we examined *Isl1* compound mutant mice carrying the *ISL*^*MN*^*: GFP-F* reporter allele in which BM and SM neurons are labeled with GFP^20^. Embryos were immunostained for GFP to detect motor neurons. Oculomotor neurons of *Isl1* mutants were defasciculated at E10.5, and became relatively normal at E11.5 (Fig. 2A–D). The mandibular branches, the motor part of the trigeminal nerve, form a thick axon bundle growing toward the target muscles with fasciculated axon tips (Fig. 2A,C,E,G). Interestingly, the trigeminal axons of *Isl1* compound mutants were defasciculated at distal axons and developed prominent extra branch in the middle of primary axon bundle (primary axon length; control, 1960.9 ± 70.0 μm; *Isl1*^*hypo/KO*^, 1506.2 ± 155.3 μm, *p* = 0.008) (length of extra branches; control, 0.0 ± 0.0 μm; *Isl1*^*hypo/KO*^, 1031.7 ± 121.5 μm, *p* < 0.001) (Fig. 2A–H,M,N, Supplementary Figure S2I). The FBM axon bundles of *Isl1* mutants were thinner and shorter (axon length; control, 1809.2 ± 85.0 μm; *Isl1*^*hypo/KO*^, 1084.4 ± 104.1 μm, *p* < 0.001) (axon thickness; control, 105.7 ± 6.5 μm; *Isl1*^*hypo/KO*^, 57.7 ± 6.5 μm, *p* < 0.001) (Fig. 2A–H,O,P, Supplementary Figure S2I). To trace inner ear efferent (IEE) projection, embryo heads were immunostained as open-book flat-mounts. IEE axons exited from the vestibular nerve root and almost reached the cochlear at E11.5 (Fig. 2Q,R)^22^. However, IEE axons in *Isl1* compound mutants were short, disrupted and hardly extended towards the inner ear (Fig. 2S,T). The number and position of somata labeled with GATA3 within the neural tube were normal in *Isl1* mutant embryos, indicating that decreased axon outgrowth in the periphery is not simply due to reduction in their cell number (control, 40.7 ± 2.3 cells; *Isl1*^*hypo/hypo*^, 45.5 ± 4.1 cells) (Supplementary Figure S3C–E)^22^. And there was no obvious sign of cell death or axon degeneration in FBM and IEE neurons of *Isl1* compound mutants since no cleaved-CASPASE-3 immunoreactivity was found in them and their explants showed robust axon outgrowth *in vitro* (neurite length; control, 1.0 ± 0.1 fold; *Isl1*^*hypo/KO*^, 1.42 ± 0.2 fold, *p* = 0.016), (neurite number; control, 16.3 ± 1.3 neurites, *Isl1*^*hypo/KO*^, 30.7 ± 2.8 neurites, *p* < 0.001) (Supplementary Figure S2A–H). The normal exit point of FBM and IEE neurons in the neural tube is at the lateral position of r4 (Fig. 2U). In E11.5 flat-mounted hindbrains of *Isl1* compound mutant mice, however, FBM and IEE axons had additional exit points at more medial positions with disorganized projections (Fig. 2V, Supplementary Figure S3B). In summary, axon pathfinding by BM neurons was disrupted when the ISL1 level was reduced.

We also examined the axon projections of SM neurons, whose identity was affected in *Isl1* compound mutant mice. In these mutants, SM axons such as those of the hypoglossal nerve (XII) were almost absent with only a few aberrant axons to be seen (Fig. 2J,L). Similar results were obtained in *Hb9::GFP* transgenic mice, in which SM neurons are selectively labeled (Fig. 2W)^8^,^10^. Peripheral axons of SM neurons were almost absent from caudal hindbrains, and a few misrouted projections remained in cervical neurons (Fig. 2X). Cell bodies disappeared and interneuron-like trajectories spanning the A-P axis of the hindbrain were visible in flat-mounted hindbrains (Fig. 2Y–BB). Thus, axon projection in SM neurons is also disrupted in *Isl1* compound mutant mice.

### *Slit2* signaling is defective in *Isl1* mutant BM neurons

**Figure 3:**
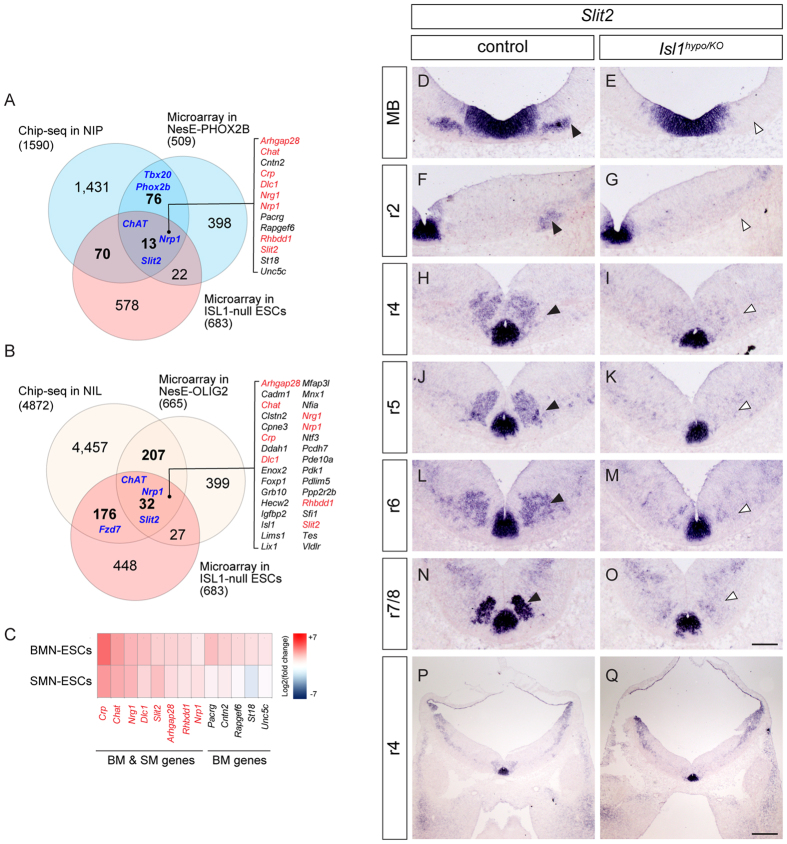
Reduced *Slit2* transcripts in *Isl1* mutant BMNs. (**A,B**) Venn diagrams indicating the overlap between ISL1 binding sites in a Chip-seq analysis and two independent microarray analyses in BM (**A**) and SM (**B**) cells. The list of genes found in both BM and SM cellular contexts is shown in red. (**C**) Heatmap of genes associated with BM and SM neurons. Red indicates higher relative expression, and blue indicates lower relative expression compared to the median values of the two groups. (**D–Q**) At E11.5, *Slit2* mRNA is undetectable in oculomotor (**E**), trigeminal (**G**), facial (**I,K,M**) and SM (**O**) neurons (white arrowheads) unlike their littermate controls (black arrowheads, **D,F,H,J,L,N**). (control: n = 3; *Isl1* mutant: n = 3; number of embryos). Scale bars: in (**O**), 100 μm for (**D–O**); in (**Q**), 200 μm for (**P,Q**).

Previously we performed microarray screens of embryonic stem cells (ESCs) derived from *Isl1* knockout cells^23^. Since ISL1 is a transactivator, we focused on 683 genes significantly downregulated in *Isl1* null cells (*P* < 0.05 with Bonferroni correction). To search for direct downstream targets of ISL1, we also re-analyzed previously published ChIP-seq data for ISL1 genomic binding sites retrieved from an ESC line, induced by NGN2, ISL1, PHOX2B (NIP) with BM/VM neuronal properties, and combined it with microarray results from *Isl1* null cells^1^. About 1,590 genes had significant binding peaks (>1.5-fold with *P* < 0.01) for ISL1 in their vicinity (within ± 2 kb of gene boundaries), and 83 of them were downregulated in *Isl1-*deficient cells. By adopting additional microarray data obtained from NesE-PHOX2B ESC line derived visceral MNs, we finally selected 13 genes as potential ISL1 targets in BM neurons; these included genes for choline acetyltransferase (*Chat*), neuropilin1 (*Nrp1*) and *Slit2* (Fig. 3A, Supplementary Table S1)^24^. ISL1 is expressed in both BM and SM neurons, therefore we investigated whether ISL1-mediated transcriptional regulation is conserved in BM and SM neurons. We analyzed another ChIP-seq and microarray datasets derived from two independent ESC lines with SM characteristics, NIL (NGN2, ISL1, LHX3) and NesE-OLIG2, which differentiated from mESCs to SM neurons by expressing OLIG2 under the *Nestin* enhancer^1^,^24^. As a result, we identified 32 genes as putative downstream genes of ISLl in SM neurons (Fig. 3B, Supplementary Table S1). To gain a better understanding of shared or cell type-specific transcriptional control of ISL1 in BM neurons, we focused on 13 genes altered in BMN-ESCs; 8 of them were altered in both BMN and SMN-ESCs (BM & SM genes) and 5 of them were only altered in BMN-ESCs (BM genes) (Fig. 3C). Thus, transcriptional control of ISL1 may be partly conserved between BM and SM neurons.

To verify whether transcript levels of genes selected by the bioinformatics analysis are actually altered in *Isl1-*deficient BM neurons, we examined mRNA or protein levels of major axon guidance genes or others including NRP1*, Slit2,* CHAT, TAG-1 and *Unc5c*. All of them were present in BM neurons, however, only *Slit2* transcript levels were diminished in BM neurons of *Isl1* mutants (Fig. 3D–Q, Supplementary Figure S4A–H). *Slit2* mRNA levels were high in wild type SM neurons and floor plates, and relatively low but definite in post-migrated cranial motor neurons, including oculomotor neurons and trigeminal and migrating FBM neurons (Fig. 3D,F,H,J,L,N,P)^25^. Remarkably, the levels of *Slit2* transcripts in oculomotor, trigeminal and FBM neurons were greatly attenuated in *Isl1* compound mutant mice, whereas *Slit2* expression in the floor plate was normal (Fig. 3E,G,I,K,M,O,Q). *Slit2* expression in SM neurons also disappeared in *Isl1* mutants since SM neurons transfate to become interneurons (Fig. 3O, see Fig. 1BB,CC). Transcripts of the related ligands *Slit1* and *Slit3,* and their receptors *Robo1* and *Robo2,* were not changed in the compound mutants nor selected as candidate genes in the bioinformatics analysis (Supplementary Figure S4I–R).

Since ROBO-SLIT signaling controls axon navigation, the extra branches in trigeminal axons of *Isl1* mutants could be due to defective ROBO-SLIT signaling^26^,^27^. In line with this, FBM somata were mispositioned when *Robo1* and *Robo2* are downregulated, which indicates that ROBO-SLIT signaling is important in developing FBM neurons^28^. We therefore examined the FBM projections in *Robo1*^*−/−*^*; Robo2*^*−/−*^ mice traced with the *ISL*^*MN*^*: GFP-F* reporter^29^. Trigeminal mandibular (V) axons of *Robo* mutants were defasciculated or had extra branches (primary trigeminal axon length; control, 944.4 ± 75.4 μm; *Robo1*^*−/−*^*; Robo2*^*−/−*^, 809.9 ± 93.2 μm) (length of extra branches; control, 0.0 ± 0.0 μm; *Robo1*^*−/−*^*; Robo2*^*−/−*^, 398.2 ± 33.2 μm, *p* < 0.001) (Supplementary Figure S5). In addition, FBM axons were thinner and shorter in *Robo* mutants, similar to axons of *Isl1* mutant mice (facial axon length; control, 1171.7 ± 49.2 μm; *Robo1*^*−/−*^*; Robo2*^*−/−*^, 976.9 ± 56.3 μm, *p* = 0.046) (facial axon thickness; control, 86.5 ± 5.1 μm; *Robo1*^*−/−*^*; Robo2*^*−/−*^, 45.1 ± 4.9 μm, *p* = 0.002) (Supplementary Figure S5). This is not due to reduced number of FBM neurons since it was reported that the number of FBM somata in *Robo1*^*−/−*^*; Robo2*^*−/−*^ were comparable^28^. Although we cannot completely exclude the possibility that other genes controlled by Isl1 are involved, projection errors found in *Isl1* compound mutant could be affected by defective ROBO-SLIT signaling at least in part.

### ISL1 controls *Slit2* transcription in SM and BM neurons together with LHX3 and LHX4

**Figure 4:**
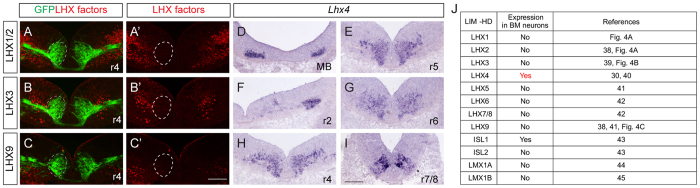
LHX4 is present in BMNs. (**A–C’**) LHX1/2, LHX3 and LHX9 are not detectable in BM neurons labeled in green in transverse sections of E11.5 hindbrains of *ISL*^*MN*^*: GFP-F* mice (white dotted lines). (**D–I**) *Lhx4* transcript is present in BM neurons of E11.5 transverse hindbrain sections. (**J**) A list of LIM-homeodomain transcription factors present in BMNs. Scale bars: in (**C’**), 100 μm for (**A–C’**) in (**I**), 100 μm for (**D–I**).

ISL1 is a LIM-HD transcription factor, whose N-terminal LIM domains mediate protein-protein interactions. In the cortex and spinal cord, ISL1 forms complexes with LHX8 and LHX3, respectively^2^,^19^. We reasoned that it may similarly require a LIM-HD transcription factor in its role in BM neurons. The only LIM-HD transcription factor known to be present in BM neurons is LHX4 (Fig. 4A–J)^30^. *Lhx4* mRNA was found to be present in oculomotor neurons, migrating and postmitotic trigeminal and facial motor neurons, and its expression nicely overlapped with *Slit2* transcripts, which made it a plausible candidate for interacting with ISL1 (Fig. 4D–I, and see Fig. 3). If LHX4 did form a complex with ISL1 in BMNs, removing *Lhx4* will also downregulate *Slit2* expression and may cause axon defects. To test this hypothesis, we examined *Slit2* mRNA expression in BM neurons of *Lhx4* knock-out mice^31^. Adjacent sections were used to locate BM neurons labeled with ISL1. In littermate controls, BM neurons were migrating from r4 to r6 and *Slit2* transcripts were found in r5 (Supplementary Figure S6A,C). However, BM neurons were located in r4-r6 of the *Lhx4* knock-out hindbrain but *Slit2* transcripts were undetectable (Supplementary Figure S6B,D). Together these results suggest that ISL1-LHX4 hexamer complexes control *Slit2* transcription in BMNs.

**Figure 5:**
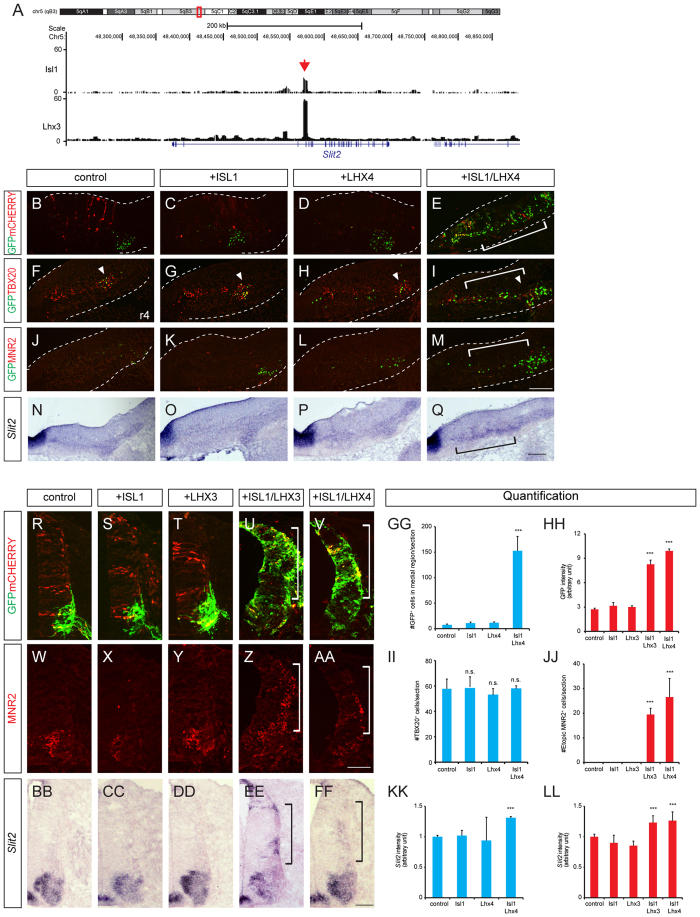
ISL1-LHX3/4 complexes activate the *Slit2* enhancer. (**A**) ChIP-seq analysis of ISL1 and LHX3 binding to the *Slit2* genome. Red arrow indicates *Slit2* enhancer. (**B–Q**) GFP expression in transverse sections of HH stage 24 chick embryos electroporated with the *Slit2* enhancer: *GFP* reporter, the *CMV::mCherry* vector as an internal control, ISL1, or LHX4 as indicated. Expression of *Slit2*, TBX20 and MNR2 was assessed in adjacent sections. In the hindbrain, the GFP signal is present in BM neurons (arrowheads), which have become medially expanded in the presence of ISL1 and LHX4 (brackets). *Slit2* mRNA is induced in the same area (bracket), while the number of TBX20-expressing cells is unchanged. (**R-FF**) In the spinal cord, GFP activity is present in SM neurons. In the presence of ISL1-LHX3 or ISL1-LHX4, however, GFP expression and *Slit2* mRNA have expanded dorsally together with ectopic MNR2-expressing cells (brackets) (>3 sections in 3 embryos in each group). (**GG–LL**) Quantification of GFP and *Slit2* intensities, and TBX20 and MNR2-expressing cells (>3 sections in 3 embryos in each group). Error bars represent SEM. ***p < 0.001; Mann-Whitney rank sum test in GG (n = 4), unpaired Student’s t-test in (**HH–LL**) (n = 4); n.s., not significant. Scale bars: in (**M**), 100 μm for (**B–M**) in (**Q**), 100 μm for (**N–Q**) in (**AA**), 100 μm for (**R-AA**) in (**FF**), 100 μm for (**BB–FF**).

We searched for ISL1 and LHX binding sites in the genomic locus of *Slit2* in the ChIP-seq data in NIL cells and chose the highest ChIP-seq peak, which was located in the 6th intron of *Slit2* (hereafter referred to as the *Slit2* enhancer) (Fig. 5A)^1^. Interestingly, ChIP-seq data for LHX3 binding loci also showed the highest peak at the same genomic region (Fig. 5A)^32^,^33^,^34^. This indicates that ISL1 and LHX3 together may drive *Slit2* transcription in SM neurons, probably by forming the ISL1 and LHX3 hexameric complex^2^,^35^. We found that a GFP reporter carrying the *Slit2* enhancer was active in both BM and SM neurons when electroporated into the hindbrains and spinal cords, respectively (Fig. 5B,R). To pinpoint the cells in which the *Slit2* enhancer was active in the hindbrain, we generated a *nucGFP* reporter and introduced it by electroporation together with *CMV::mCherry* as an internal control. When ISL1 or LHX4 was introduced by electroporation, the nucGFP signal was mostly confined to the FBM nucleus, as in the control group (control, 7.7 ± 1.5 cells; ISL1, 10.9 ± 2.0 cells; LHX4, 11.8 ± 1.6 cells) (Fig. 5C,D,GG). When both ISL1 and LHX4 were introduced, the number of GFP-expressing cells medial to the facial nucleus increased in the hindbrains (ISL1 + LHX4, 152.8 ± 28.1 cells, *p* < 0.001) (Fig. 5E,GG). More importantly, in this medial region, upregulation of ck*Slit2* transcript was observed, indicating that ISL1 and LHX4 not only activate the *Slit2* enhancer but can also drive ectopic expression of *Slit2* mRNA (arbitrary unit of *Slit2* intensity; control, 1.0 ± 0.0 fold; ISL1, 1.0 ± 0.1 fold; LHX4, 0.9 ± 0.4 fold; ISL1 + LHX4, 1.3 ± 0.0 fold, *p* < 0.001) (Fig. 5Q,KK). This was not due to ectopic production of BM neurons since no additional BM neurons labeled with TBX20 were found in this region (control, 57.8 ± 7.75 cells; ISL1, 58.0 ± 8.0 cells; LHX4, 52.8 ± 5.1 cells; ISL1 + LHX4, 57.8 ± 2.4 cells) (Fig. 5I,M,II). We observed similar effects with ISL1 and LHX3 in SM neurons: ISL1 and LHX3 synergized to expand GFP activity to the dorsal spinal cord where additional MNR2 (ISL1 + LHX3, 19.5 ± 2.5 cells, *p* < 0.001) and ck*Slit2* mRNAs were found, while ISL1 or LHX3 alone did not (arbitrary unit of *Slit2* intensity; control, 1.0 ± 0.0 fold; ISL1, 0.9 ± 0.1 fold; LHX4, 0.9 ± 0.1 fold; ISL1 + LHX3, 1.2 ± 0.1 fold; ISL1 + LHX4, 1.3 ± 0.1 fold, *p* < 0.001) (Fig. 5R–U,W–Z,BB–EE,HH,JJ,LL). LHX4 had similar effect to LHX3 (ISL1 + LHX4, 26.6 ± 7.5 cells, *p* < 0.001), indicating that the two LHX factors may play redundant roles in SM neurons (Fig. 5V,AA,FF). We conclude that ISL1 and LHX3 are sufficient to generate SM neuronal traits and induce *Slit2* transcription in the spinal cord.

**Figure 6:**
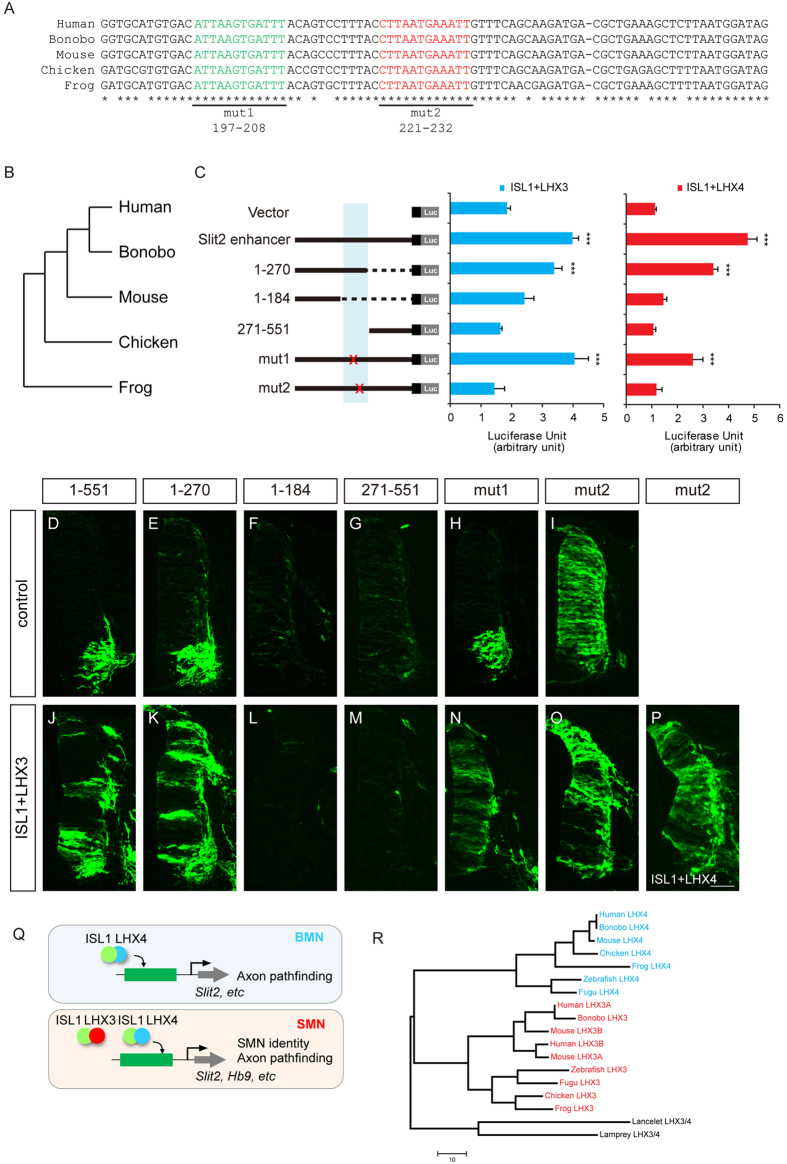
ISL1-LHX3 and ISL1-LHX4 binding sites in the *Slit2* enhancer are evolutionarily conserved. (**A**) Multiple sequence alignment of potential LIM-HD-binding sites (green and red texts) in the *Slit2* enhancer in human, bonobo, mouse, chicken and frog. Point mutations introduced were shown below. (**B**) Phylogenetic tree of species in which *Slit2* enhancers were analyzed. (**C**) *Slit2* enhancer luciferase activity was measured in HEK 293T cells. Error bars represent s.e.m. ***p < 0.001, unpaired Student’s t-test (n > 3). (**D–P**) Activity of *Slit2* GFP reporter derivatives measured by *in ovo* electroporation of chicks. MN-specific GFP activity was present in reporters with regions 1–551, 1–270 and mut1 but not in those with regions 1–184, 271–551 and mut2 (>11 sections in 3 embryos in each group). (**Q**) Model of the regulation of transcription regulation of the ISL1 and LHX factors during motor neuron development. The diagram of the ISL1-LHX hexamer complex is simplified. (**R**) Phylogenetic tree of species for which LHX3 and LHX4 protein sequences were analyzed. Scale bar: in (**P**), 100 μm for (**D–P**).

Since LIM-HD transcription factors bind to AT-rich sequences, we searched for binding sites of ISL1 and LHX3/4 in the *Slit2* enhancer^1^,^36^,^37^. There were a few AT-rich motifs in the *Slit2* enhancer and the enhancer was evolutionarily conserved in different species (Fig. 6A,B). When a luciferase reporter with the full length *Slit2* enhancer 1–551 was transfected into HEK 293T cells, ISL1 and LHX3 (3.98 ± 0.20 fold, *p* < 0.001) and ISL1 and LHX4 (4.74 ± 0.37 fold, *p* < 0.001) induced strong transactivation of the reporter (Fig. 6C). When the activity of reporters with deletions was measured, only a reporter carrying region 1–270 (ISL1 + LHX3, 3.38 ± 0.26 fold; ISL1 + LHX4, 3.41 ± 0.17 fold, *p* < 0.001) but not ones carrying regions 1–184 or 271–551 was activated by ISL1-LHX4 or ISL1-LHX3, indicating that the ISL1-LHX3/4 binding sites lie within region 184–270. There are two AT-rich motifs within this region; we therefore mutated them individually to produce reporters mut1 and mut2 (Fig. 6C). Mut1 reporter activity was induced by ISL1-LHX3/4 (ISL1 + LHX3, 4.04 ± 0.46 fold; ISL1 + LHX4, 2.61 ± 0.39 fold, *p* < 0.001), but that of mut2 was not, indicating that ISL1-LHX3/4 binds to the region containing the mut2 site (Fig. 6C). We next introduced GFP reporters with deletions or point mutations into the spinal cord to test whether these reporters retained motor neuron-specific expression or not. Paralleling the results of the luciferase assays, we found that the reporters harboring regions 1–551 and 1–270, and the mut1 reporter produced normal motor neuron-specific GFP activity and were induced by ISL1-LHX3 (Fig. 6D,E,H,J,K,N). On the other hand, the reporters harboring regions 1–184 and 271–551 generated very little GFP and were not induced by ISL1-LHX3, confirming that they lacked the transcription factor binding sites essential for reporter activity (Fig. 6F,G,L,M). The mut2 reporter had also lost motor neuron-specific activity and was not induced by ISL1-LHX3 or ISL1-LHX4 (Fig. 6I,O,P). Together, these results show that the *Slit2* enhancer is activated by ISL1 and LHX4 in BM neurons, and ISL1 and LHX3 in SM neurons (Fig. 6Q).

## Discussion

Cranial motor neurons are divided into two subpopulations called BM/VM neurons and SM neurons with different origins and properties^5^. In this study, we demonstrated that BM/VM neurons did arise in *Isl1* mutants but their BM neurons were defective to varying degrees in cell body migration and axon projection, supporting their combinatorial action.

ISL1 requires additional LIM-HD factor to form a multimeric complex for its function as demonstrated in the cortex (with LHX8) and spinal cord (with LHX3), which may serve different roles^2^,^19^,^35^. Among total 12 members, we speculated that LHX4 is the plausible LIM-HD transcription factor that works with ISL1 in BM neurons. LHX4 is present in both BM and SM neurons, while LHX3 is only present in SM neurons^30^,^38^,^39^,^40^,^41^,^42^,^43^,^44^,^45^. The presence of LHX4 in motor neurons has been known for a while but its role has been relatively underestimated due to its redundancy with LHX3 in SM neurons; only when both LHX3 and LHX4 are eliminated, SM neurons transfated to interneurons^31^,^39^,^46^,^47^. Interestingly, BM neurons such as spinal accessary motor column (SAC) cells remain in the absence of LHX3 and LHX4, which indicates that specification of BM neurons is intact without LHX4^39^. We also observed that BM neurons were normally specified in the absence of ISL1, which together suggests that LHX4 is dispensable for specification of BM neurons. In this study, we demonstrated that both LHX3 and LHX4 have equivalent abilities to induce ectopic SM neurons when electroporated with ISL1 in the chick spinal cord. However, forced expression of ISL1 and LHX4 did not induce ectopic BM neurons or SM neurons in r4 chick hindbrains, indicating that potential of progenitors are already regionally-specified. Genome-wide analyses to distinguish BM and SM neuronal populations demonstrated that genome-wide binding sites of ISL1 differ in ESC-derived BM and SM neurons^1^,^24^. Moreover, ISL1 tends to bind adjacent to PHOX2 or LHX3 in BM and SM neurons, respectively^1^,^24^. Thus, different transcription profiles and environmental factors segregates BM and SM neurons, in which LHX4 behaves differently in cell fate and axon development^2^,^8^.

Several lines of evidence suggest that LIM codes control various functions in differentiating neurons, since the positioning of cell bodies, axon projections, neurotransmitter identities and ion channel expression, are all affected in many different neurons when ISL1 is downregulated^9^,^23^,^48^,^49^,^50^,^51^,^52^. Both ISL1 and LHX4 appeared to be required to induce *Slit2* transcription as shown in cell lines, chick and mouse embryos, which favors the idea that they constitute the LIM code. Moreover we were able to locate their binding region in the intron of the *Slit2* locus within the *Slit2* enhancer. The activity of the *Slit2* enhancer was highest only when both factors were present and mutating the putative binding sites abolished responsiveness. Furthermore, misexpression of ISL1 and LHX3/4 induced ectopic *Slit2* transcription, raising the possibility that *Slit2* is a direct target of ISL1 and LHX3/4. The molecular and genetic evidence that we have obtained indicates that different LIM codes assign different functions; BM neurons utilize the ISL1-LHX4 complex for axon development, while SM neurons employ ISL1-LHX3 and ISL1-LHX4 complexes to assign cell identity.

LHX4 is highly homologous to LHX3, with 66% identity at the amino acid level and 95% identity in the homeodomain, and they have similar bioactivity *in vitro*^18^,^53^,^54^,^55^. However, high resolution analysis of sequence preference revealed that, although the homeodomains of LHX3 and LHX4 have generally similar binding preferences, they have slightly different preferences for weaker motifs^56^. In addition, their differences in the LIM domain (about 82%), the interface that binds to other proteins, imply that subtle differences in this region may lead to divergence of their functions^18^. Previously we and others demonstrated that transcription is regulated differently in hindbrain and spinal motor neurons and is correlated with motor neuron diversification during evolution^57^,^58^. Thus, it is possible that LHX3 and LHX4 have evolved to share both common and distinct roles. They diverged recently during evolution: only one LHX3/4 factor is found in the lancelet and lamprey genome, in both of which hindbrain patterning is not yet fully established^59^ (Fig. 6R). The lancelet lacks a hindbrain, and in the lamprey the position of BM nuclei do not match with specific rhombomeres^57^,^60^,^61^. However, LHX3 and LHX4 have diverged in more advanced aquatic vertebrates in which hindbrain patterning is complete, i.e., BM nuclei align with rhombomere boundaries^57^. Thus, the segregation of LHX3 and LHX4 during evolution may suggest that different LHX factors serve different functions in SM and BM neurons.

In attempts to dissect out the genetic programs that define BM and SM neurons, several groups have compared the gene expression profiles of ESCs fated to become BM vs. SM neurons and found a large number that were either differentially enriched or similarly expressed in the two cell types^1^,^24^,^35^. For instance, a genome-wide Chip-seq analysis revealed that about 22–26% of genes were bound by ISL1 in both cell types while the rest were bound by ISL1 in only either of them^1^. However, when compared with microarray results from *Isl1-*deficient cells, only less than 50 genes were predicted to be targets of ISL1. When we examined mRNA expression of candidate genes by *in situ* hybridization, only transcript level of *Slit2* transcripts was significantly reduced in BM neurons of *Isl1* mutant mice. Nevertheless, more genes are likely to be controlled by Isl1, given the number of genes bound by Isl1 in ChIP-Seq analysis (1,590 genes) and the number of genes (683 genes) whose expression level was altered in our microarray results^62^. One possibility is that embryonic stem cells used in most studies may not fully represent cellular context of motor neurons *in vivo*, or *in situ* hybridization technique is not sensitive enough to detect subtle differences in gene expression level. More comprehensive transcriptome analysis *in vivo* will give us better clues to understand full repertoire of target genes controlled by ISL1 in developing motor neurons.

ROBO-SLIT signaling serves various developmental roles in the CNS including axon guidance, neuronal migration and axon and dendritic branching, mostly ‘within’ the neural tube. For instance, in the spinal cord, cell bodies and axons of motor neurons cross the midline when *Slit* or *Robo* genes are downregulated^28^,^63^. Similarly, in the hindbrain, errors in central projection and migration of BM neurons have been reported in *Slit* and *Robo* mutants: IEE neurons fail to cross the midline and trigeminal axons project in ectopic locations, and cell bodies of FBM neurons abnormally cross the midline^25^,^28^. However, the role of ROBO-SLIT signaling in peripheral projections is still less understood. It is reported that ROBO receptors control on peripheral projections of BM/VM neurons and their neurites respond to SLIT ligands *in vitro*^25^. This is reminiscent of ROBO-SLIT signaling in spinal cord motor axons; both SLIT2 and ROBO receptors are present in motor axons and transmit ROBO-SLIT signaling in an autocrine/juxtaparacrine manner^64^. It is still uncertain by which mechanisms *Slit2* expression in them influence axons of BM/VM neurons. There are several locations in the developing CNS in which a ligand and its receptor co-exist within the same population^65^,^66^,^67^,^68^. For instance, altering intrinsic SEMA3A levels affected motor axon trajectories and the sensitivity of growth cones to exogenous semaphorins, probably because the endogenous ligand masked the receptor on the cell surface or influenced its trafficking^69^. Alternatively, the presence of a ligand may affect receptor activity, i.e., phosphorylation or downstream signaling, as shown in EPHRIN/EPH and SEMA6A/PLEXINA4 interactions^66^,^68^. It is not clear whether SLIT ligands control the availability of ROBO receptors or influence downstream pathways shared with other navigation cues. Nevertheless, our results demonstrate that ISL1 and LHX factors control motor neuron identity and *Slit2*-transcription in a cell-type-specific manner.

## Methods

### Mice

*Isl1* hypo, *Isl1* null, *Isl1* flox mice, *ISL*^*MN*^*: GFP-F*, and *Hb9::GFP* mice were described previously^8^,^10^,^14^,^35^,^63^. *Nestin-Cre* mice were obtained from Jackson laboratory. Wildtype C56BL/6 mice (6–8 weeks old) were purchased from Damul Science. All experiments used protocols approved by the Animal Care and Ethics Committees of the Gwangju Institute of Science and Technology (GIST) in accordance with the National Institutes of Health Guide for the Care and Use of Laboratory Animals. The day when a vaginal plug was detected was designated embryonic day 0.5 (E0.5).

### DNA constructs

Mouse *Slit2* enhancer (chr5:48181677-48182228) was amplified by PCR using the genomic DNA from mouse. PCR fragments were subcloned into the *pCS2 mini CMV-GFP* and *pCS2 mini CMV-nucGFP* which contains a 60 bp TATA box and the transcription initiation site of the cytomegalovirus (CMV) promoter. The *mini CMV* promoter and *EGFP* sequences were amplified by PCR from *pEGFP-N1* (Clontech). For luciferase assay, *Slit2* enhancer PCR fragments were subcloned into the *tk-luciferase* reporter vector (Clontech). *Isl1* and *Lhx3* plasmids were described previously^8^. Mouse *Lhx4* was amplified by PCR using mouse cDNA and subcloned into the *pCAGGS1* vector. Fragments of mouse *Slit2* enhancer 1–270, 1–184, 271–551 were amplified by PCR and mutations were introduced in mouse *Slit2* enhancer by PCR-based mutagenesis.

### *In ovo* electroporation

*Slit2* enhancer::GFP, *Slit2* enhancer::nucGFP, pCAGGS1-mIsl1, pCAGGS1-mLhx3, pCAGGS1-mLhx4 and pmCherry-C1 (Clontech) were electroporated into the chick hindbrains and spinal cords at HH stages 10 to 12, using a square wave electroporator (BTX) with 5 pulses of 18 V, 50 ms at 1 s intervals. Embryos were harvested at HH stages 23 to 24.

### Luciferase assays

HEK 293T cells were seeded and incubated for 24 hours, and transiently transfected with reporters and transcription factors using Lipofectamine 2000 reagent (Invitrogen). *CMV-β-galactosidase* plasmid was co-transfected to normalize transfection efficiency. Cells were harvested about 40 hours after transfection, and cell extracts were assayed for luciferase assays and β-galactosidase assays. Data represent as means of triplicate value and all transfections were repeated independently at least three times.

### Immunohistochemistry and *In situ* hybridization

Immunohistochemistry or *in situ* hybridization was performed as described previously^8^. The following antibodies were used: rabbit and guinea pig anti-HB9^70^, rabbit anti-GFP (Invitrogen), mouse anti-GFP (Sigma), guinea pig anti-CHX10^2^, rabbit anti-TBX20^71^, rabbit anti-PHOX2B^72^, rabbit anti-ISL1/2^10^, rabbit anti-LHX1/2^10^, guinea pig anti-LHX3^73^, goat anti-LHX9 (Santa Cruz Biotechology). For flat-mount or whole mount immunostaining, flat-mounted hindbrains or embryos were fixed in 4% PFA, permeablized and then processed for immunostaining^71^. For *in situ* hybridization, embryonic mouse cDNA at E11.5 was used to generate riboprobes using an Advantage cDNA PCR kit (Clontech).

### Quantification

To quantify ISL1 immunoreactivity in FBM neurons, 12 μm-thick transverse sections of r4 hindbrains were immunolabeled with anti-ISL1/2 and PHOX2B antibodies. The positions of *Isl1-*null FBM neurons were identified from the expression of PHOX2B. The background-subtracted pixel intensities of FBM nuclei were measured using MetaMorph software (Molecular Devices). To count the number of BM and SM neurons in each rhombomere, at least 3 sections from 3 embryos were analyzed for each group. The numbers of GFP, MNR2 and TBX20-expressing cells were determined in 12 μm-thick transverse sections after immunohistochemical staining. The number of nucGFP-expressing cells in the hindbrain was measured in the region medial to FBM nucleus in r2 and r4, as defined by TBX20 expression in the same sections. The level of *ckSlit2* transcripts in the medial region of hindbrain and 250 × 650 pixel areas in the dorsal spinal cord were measured in adjacent transverse sections using ImageJ software. In the spinal cord, the background-subtracted pixel intensities of GFP in 250 × 650 pixel areas in the dorsal spinal cord were measured using ImageJ. At least 9 sections from 3 chick embryos were analyzed in each group. Statistical significance was analyzed by unpaired Student’s t-test or Mann-Whitney rank sum test as indicated in figure legends. To measure the length of trigeminal and FBM axons, peripheral projections from the exit point to the nerve terminals were manually traced. To calculate mean axon thickness, axon bundles were divided into 5 parts from proximal to distal and thickness at each intersection was averaged. All quantifications in images were analyzed in MetaMorph software (Molecular Devices).

### Bioinformatic analysis

Enhancer sequences from human, bonobo, mouse, chicken and frog were retrieved from UCSC genome browser and aligned with mVISTA (genome.lbl.gov/vista) using the LAGAN alignment tool. LHX3/4 protein sequences from human, bonobo, mouse, chicken, frog, zebrafish, fugu, and lancelet were retrieved from NCBI protein database and lamprey sequence was retrieved from Japanese lamprey genome database. The LHX3/4 evolutionary history was inferred using the maximum parsimony method. The percentage of replicate trees in which the associated taxa clustered together in the bootstrap test (1,000 replicates) are shown below the branches. The MP tree was obtained using the Subtree-Pruning-Regrafting (SPR) algorithm. The tree is drawn to scale, with branch lengths calculated using the average pathway method and are in the units of the number of changes over the whole sequences. Evolutionary analyses were conducted in MEGA6. For microarray analysis, we considered gene expression change as significant if the change of expression was ≥2 fold in genes down-regulated in *Isl1-*deficient ESC-derived motor neurons and ≥2.5 fold in genes induced in NesE-PHOX2B ESC-derived VM neurons and NesE-OLIG2 ESC-derived SM neurons^23^,^24^. To search for direct downstream targets of ISL1, we selected up to 1.5-fold binding peaks within ± 2 kb of gene boundaries in previously published dataset^1^.

## Additional Information

**How to cite this article:** Kim, K.-T. *et al*. ISL1-based LIM complexes control *Slit2* transcription in developing cranial motor neurons. *Sci. Rep.* **6**, 36491; doi: 10.1038/srep36491 (2016).

**Publisher’s note:** Springer Nature remains neutral with regard to jurisdictional claims in published maps and institutional affiliations.

## Supplementary Material

Supplementary Information
