# Supplementary material for: ISL1-based LIM complexes control Slit2 transcription in developing cranial motor neurons
Source: Sci Rep. 2016 Nov 7;6:36491. doi: 10.1038/srep36491 (PMC5098159; doi:10.1038/srep36491)
Supplement: Supplementary Information [file srep36491-s1.pdf]

Supplementary information

ISL1-based LIM complexes control *Slit2* transcription in developing cranial motor neurons

Kyung-Tai Kim[1], Namhee Kim[1], Hwan-Ki Kim[2], Hojae Lee[1], Hannah N. Gruner[3],

Peter Gergics[4], Chungoo Park[5], Grant S. Mastick[3], Hae-Chul Park[2] and Mi-Ryoung Song[1,*]

[1]School of Life Sciences, Gwangju Institute of Science and Technology, Oryong-dong, Buk-gu, Gwangju 500-712, Republic of Korea

[2]Graduate School of Medicine, Korea University, Ansan 425-707, Korea

[3]Department of Biology, University of Nevada, Reno, NV 89557, USA

[4]Department of Human Genetics, University of Michigan, 1241 Catherine St, Ann Arbor, Michigan, MI-48109, USA

[5]School of Biological Sciences and Technology, Chonnam National University, 77 Yongbong-ro, Buk-gu, Gwangju 500-757, Republic of Korea

*Corresponding author

email: msong@gist.ac.kr

phone: +82-62-715-2508

FAX: +82-62-715-2484

Supplementary Figure Legends

Supplementary Figure S1. Specification of BM/VM neurons is normal in *Isl1*$^{hypo/KO}$ mice.

(A-N) Distribution of BM/VM neurons labeled by the presence of *delta-Isl1*, *Tbx20* and *Phox2a* transcripts and TBX20 protein is comparable in r2 and r4 *Isl1*$^{hypo/KO}$ hindbrains. (O-R) The level of *delta-Isl1* transcripts and TBX20 protein in *Isl1* mutant FBM neurons is reduced in r5. Note that the FBM somata of *Isl1*$^{hypo/KO}$ mice spread more laterally (arrowheads, L, R). Scale bar: in R, 100 μm for A-R.

Supplementary Figure S2. No obvious cell death or degeneration in *Isl1* mutant motor neurons and explants.

(A-D) Cleved-CASPASE-3 immunoreactivity was not observed in E11.5 *Isl1* mutant FBM neurons (control: n = 4; *Isl1* mutant: n= 3). (E-F') Representative examples of FBM explants. (G, H) Quantification of GFP^{+} axon length and axon bundles with a diameter over 5 μm in wild-type and *Isl1* mutant explants (control: n = 9; *Isl1* mutant: n= 3). To quantify total neurite length in explant culture, total *Isl1*^{MN}*;GFP-F* fluorescent axons area (#pixels) were measured. The number of axon fasciculation was determined by a diameter over 5 μm axons for each explant. (*$p < 0.05$, ***$p < 0.001$, unpaired Student's t-test). (I) Quantification of axon phenotype of *Isl1* compound mutant mice. (*$p < 0.05$, ***$p < 0.001$, unpaired Student's t-test). Scale bars: in D, 100 μm for A-D; in F, 200 μm for E, F; in F', 100 μm for E', F'.

Supplementary Figure S3. Inner ear efferent projection is affected in *Isl1* mutant mice.

(A-D') Immunostaining of GFP and IEE neuronal marker GATA3 in E11.5 hindbrains. GFP axons have additional exit points in *Isl1* mutant mice (arrowhead). (E) Quantification of GATA3-expressing cells. n.s.; not significant, unpaired Student's t-test (control: n = 6; *Isl1* mutant: n= 4). Scale bar: in A, 200 μm for A-B; in D', 100 μm for C-D'.

Supplementary Figure S4. Expression of candidate target genes of ISL1 and the ROBO-SLIT signaling components.

(A-H) Expression levels of candidate target genes of ISL1 including CHAT, NRP1 (BM/SM genes), TAG-1, *Unc5c* (BM genes), and NRP1 immunoreactivity was developed with DAB. (I-R) Expression levels of *Robo1, Robo2, Slit1, Slit3* are unchanged in the *Isl1* mutants. Scale bars: in H, 100 μm for A-H; in R, 100 μm for I-R.

Supplementary Figure S5. Misprojection of trigeminal and facial BM neurons when ROBO-SLIT signaling is disrupted.

(A-F) Whole mount GFP immunostaining of E10.5 (A-C) and E11.5 (D-F) ISL^{MN}*:GFP-F* embryos. Two different embryos at each stage are shown as examples of $Robo1^{-/-};Robo2^{-/-}$ mice. Trigeminal mandibular (V) nerves developed ectopic branches or defasciculated (arrowheads) and FBM (VII) axons were fewer and defasciculated in $Robo1^{-/-};Robo2^{-/-}$ mice (arrows). (G-J) Quantification of nerve length and thickness of E10.5 trigeminal and/or E11.5 facial axons as described in methods. Error bars represent s.e.m. (control: n = 5; $Robo1^{-/-};Robo2^{-/-}$ mice: n = 3; number of embryos), unpaired Student's t-test, **p* < 0.05, ****p* < 0.001, n.s.; not significant. Scale bars: in C, 200 μm for A-C; in F, 100 μm for D-F.

Supplementary Figure S6. Reduced *Slit2* transcripts in *Lhx4* KO BMNs.

(A-B') Immunostaining of ISL1 in sagittal sections of E11.5 hindbrains. Dotted lines mark ISL1-expressing BM neurons. (C-D') Fluorescence *in situ* hybridization of *Slit2* transcripts in adjacent sections. *Slit2* transcripts were detected in migrating BM neurons in r5 of control embryos, but not in BM neurons of the *Lhx4* KO embryos. Asterisk denotes non-specific auto-fluorescence in the area of damage. (E) Schematic sagittal view of the E11.5 mouse brain. tel, telencephalon; mes,

mesencephalon; rho, rhombencephalon; cb, cerebellum; is, isthmus. Scale bars: in D, 100 in μm for A-D; in D’, 100 μm for A’-D’.

Supplementary Table S1. Differentially expressed genes in *Isl1* null ES cells.

Gene	Gene symbol	Accession Number	Fold change (wild-type /*Isl1* null)
visual system homeobox 2	*Vsx2 (Chx10)*	NM_007701	0.119
gastrulation brain homeobox 2	*Gbx2*	NM_010262	0.195
plexin B3	*Plxnb3*	NM_019587	0.231
neurogenin 2	*Neurog2*	NM_009718	0.231
short stature homeobox 2	*Shox2*	NM_013665	0.265
regulator of G-protein signaling 4	*Rgs4*	NM_009062	0.271
netrin G1	*Ntng1*	NM_001163348	0.285
inhibitor of DNA binding 2	*Id2*	NM_010496	0.320
POU domain, class 3, transcription factor 3	*Pou3f3*	NM_008900	0.362
plexin C1	*Plxnc1*	NM_018797	0.403
cellular retinoic acid binding protein I	*Crabp1*	NM_013496	0.459
early B-cell factor 1	*Ebf1*	NM_007897	0.466
early B-cell factor 3	*Ebf3*	NM_001113414	0.470
neurogenic differentiation 4	*Neurod4*	NM_007501	0.481
forkhead box P1	*Foxp1*	NM_001197321	1.737
motor neuron and pancreas homeobox 1	*Mnx1 (Hb9)*	NM_019944	**1.915**
quaking	*Qk*	NM_001159516	1.949
runt-related transcription factor 1; translocated to, 1 (cyclin D-related)	*Runx1t1*	NM_001111026	1.970
choline acetyltransferase	*Chat*	NM_009891	2.211
frizzled class receptor 7	*Fzd7*	NM_008057	2.123
Eph receptor A5	*Epha5*	NM_007937	2.202
Neogenin	*Neo1*	NM_001042752	2.283
amyloid beta precursor protein (cytoplasmic tail) binding protein 2	*Appbp2*	NM_025825	2.285
actin-binding LIM protein 1	*Ablim1*	NM_001103177	2.368
REST corepressor 3	*Rcor3*	NM_144814	2.437
Kruppel-like factor 3 (basic)	*Klf3*	NM_008453	2.447
unc-5 homolog C	*Unc5c*	NM_009472	2.497
LIM and SH3 protein 1	*Lasp1*	NM_010688	2.646
very low density lipoprotein receptor	*Vldlr*	NM_001161420	2.668
cadherin 6	*Cdh6*	NM_007666	2.860
nuclear factor I/A	*Nfia*	NM_001122952	3.411
insulin-like growth factor binding protein 5	*Igfbp5*	NM_010518	3.434
inhibitor of DNA binding 4	*Id4*	NM_031166	4.345
neuropilin1	*Npn1*	NM_008737	4.660
ISL1 transcription factor, LIM/homeodomain	*Isl1*	NM_021459	**5.828**
slit homolog 2	*Slit2*	NM_178804	**6.254**

Supplementary experimental procedures

Mice

Robo1$^{-/-}$;*Robo2*$^{-/-}$ and *Lhx4*$^{-/-}$ mice were described previously[1,2]. All experiments used protocols approved by the University of Nevada, Reno Institutional Animal Care and Use Committee or the University of Michigan University Committee on Use and Care of Animals. The day when a vaginal plug was detected was designated embryonic day 0.5 (E0.5).

Immunohistochemistry and *in situ* hybridization

The following antibodies were used: anti-GATA3 (Santa Cruz Biotechnology), anti-ChAT (Chemicon), anti-NRP1 (Sigma), anti-TAG-1 (DHSB), anti-TBX20[3], anti-cleaved-CASPASE-3 (Cell Signaling). For DAB staining, DAB peroxidase substrate kit (Vector) was used. Paraffin embedded *Lhx4* knock-out and their littermate samples were boiled for 10 minutes in 0.01 M citric acid (pH 6.0) for antigen retrieval and immunoreactivity was amplified by TSA plus fluorescence kit (PerkinElmer)[4]. DNAs to generate riboprobes for *delta-Isl1*[5], *Tbx20, Phox2a, Lhx4, Robo1, Robo2, Slit1, Slit3, Unc5c* were generated from E11.5 mouse cDNAs. Fluorescence *in situ* hybridization was performed using anti-digoxigenin-POD (Roche) and TSA plus fluorescence kit (PerkinElmer).

In vitro explant culture of FBM axons

GFP-labeled FBM neurons in r4 including the floor plate were dissected under Leica fluorescence stereo-microscope. Explants were placed into the rat tail type I collagen/Magrigel mixture (BD Sciences) and cultured in MN medium (Neurobasal media containing B27 supplement, 2 mM L-glutamine, 25 mM L-glutamate, and 1% penicillin/streptomycin (Invitrogen)) for 18 h[6].

References

1 Li, H. *et al.* Gsh-4 encodes a LIM-type homeodomain, is expressed in the developing central nervous system and is required for early postnatal survival. *EMBO J* **13**, 2876-2885 (1994).

2 Chen, Z., Gore, B. B., Long, H., Ma, L. & Tessier-Lavigne, M. Alternative splicing of the Robo3 axon guidance receptor governs the midline switch from attraction to repulsion. *Neuron* **58**, 325-332 (2008).

3 Song, M. R. *et al.* T-Box transcription factor Tbx20 regulates a genetic program for cranial motor neuron cell body migration. *Development* **133**, 4945-4955 (2006).

4 Gergics, P., Brinkmeier, M. L. & Camper, S. A. Lhx4 deficiency: increased cyclin-dependent kinase inhibitor expression and pituitary hypoplasia. *Mol Endocrinol* **29**, 597-612 (2015).

5 Pfaff, S. L., Mendelsohn, M., Stewart, C. L., Edlund, T. & Jessell, T. M. Requirement for LIM homeobox gene Isl1 in motor neuron generation reveals a motor neuron-dependent step in interneuron differentiation. *Cell* **84**, 309-320 (1996).

6 Bonanomi, D. *et al.* Ret is a multifunctional coreceptor that integrates diffusible- and contact-axon guidance signals. *Cell* **148**, 568-582 (2012).

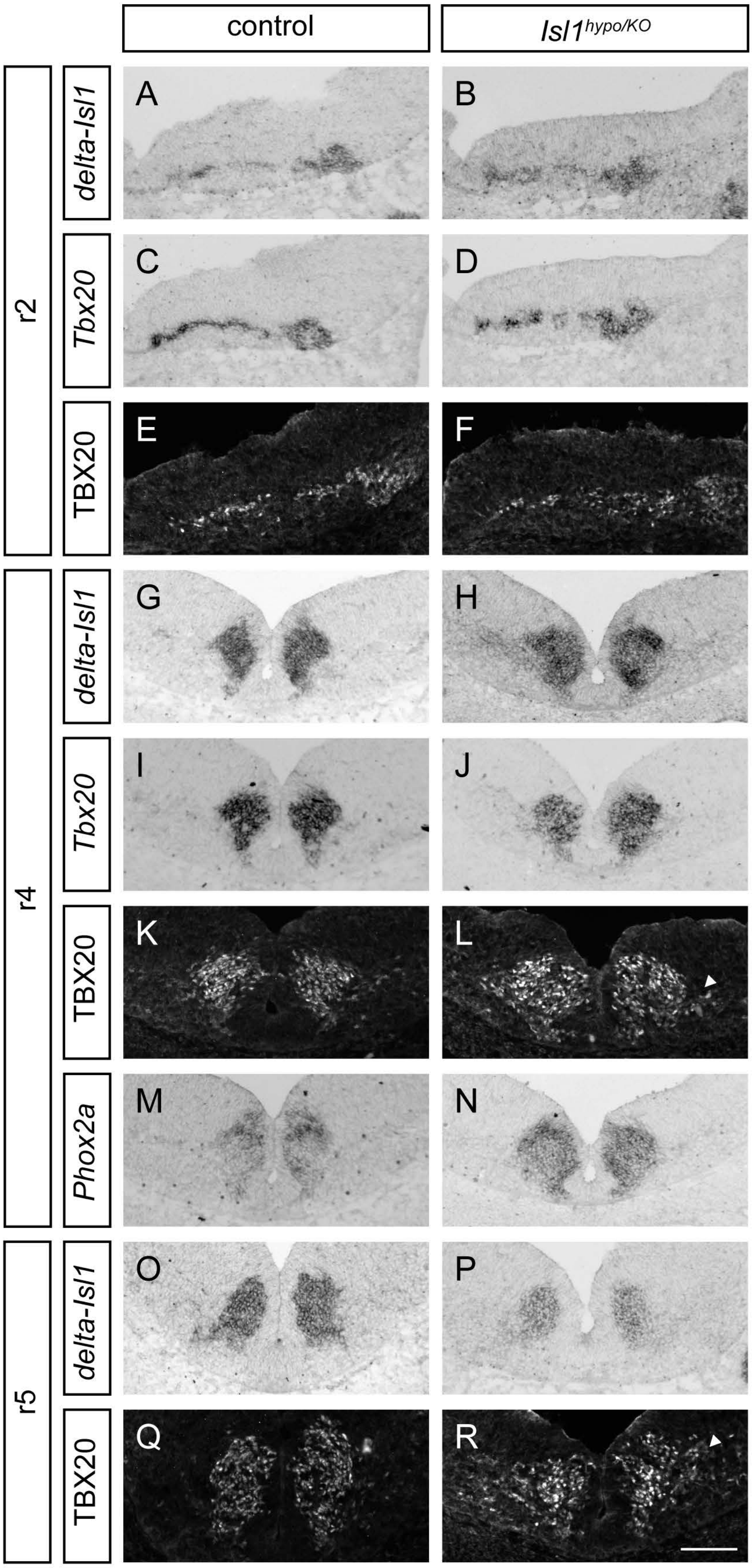
control
Isl1 hypo/KO
r2
r4
r5
delta-Isl1
Tbx20
TBX20
delta-Isl1
Tbx20
TBX20
Phox2a
delta-Isl1
TBX20
A
B
C
D
E
F
G
H
I
J
K
L
M
N
O
P
Q
R

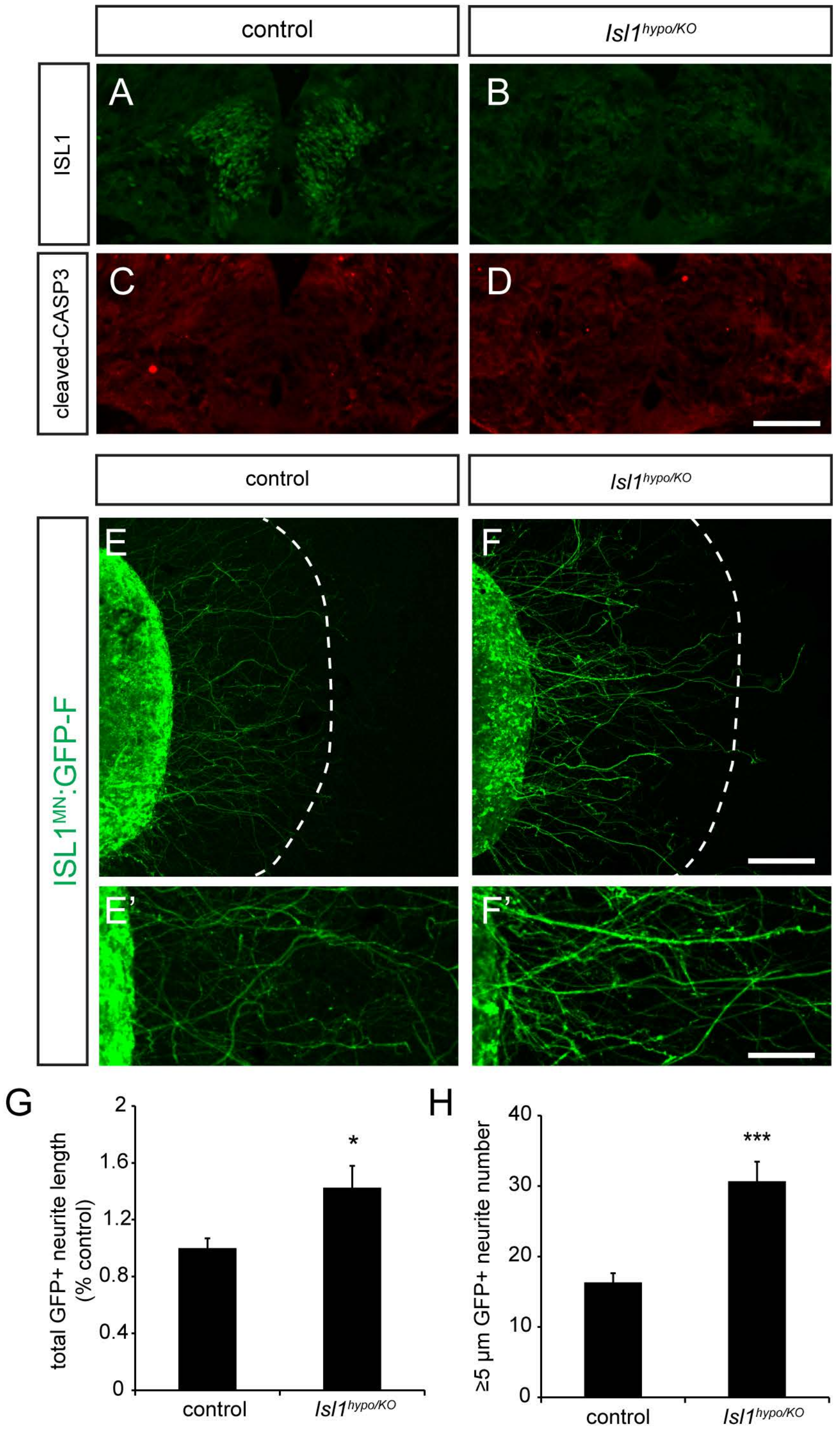

I

Stage	group	V		VII		XII
		axon length	extra branch	axon length	axon thickness	absent
E10.5	control (n=4)	949.1 ± 21.9 μm	0/4 (0%)	574.3 ± 17.7 μm	47.7 ± 3.3 μm	0/4 (0%)
	Isl1$^{hypo/KO}$ (n=3)	558.1 ± 135.0 μm*	2/3 (66%)	473.8 ± 35.8 μm*	32. 8 ± 2.0 μm*	3/3 (100%)
E11.5	control (n=11)	1960.9 ± 70.0 μm	0/11 (0%)	1809.2 ± 85.0 μm	105.7 ± 6.5 μm	0/11 (0%)
	Isl1$^{hypo/KO}$ (n=7)	1506.2 ± 155.3 μm*	7/7 (100%)***	1084.4 ± 104.1 μm***	57.7 ± 6.5 μm***	7/7(100%)

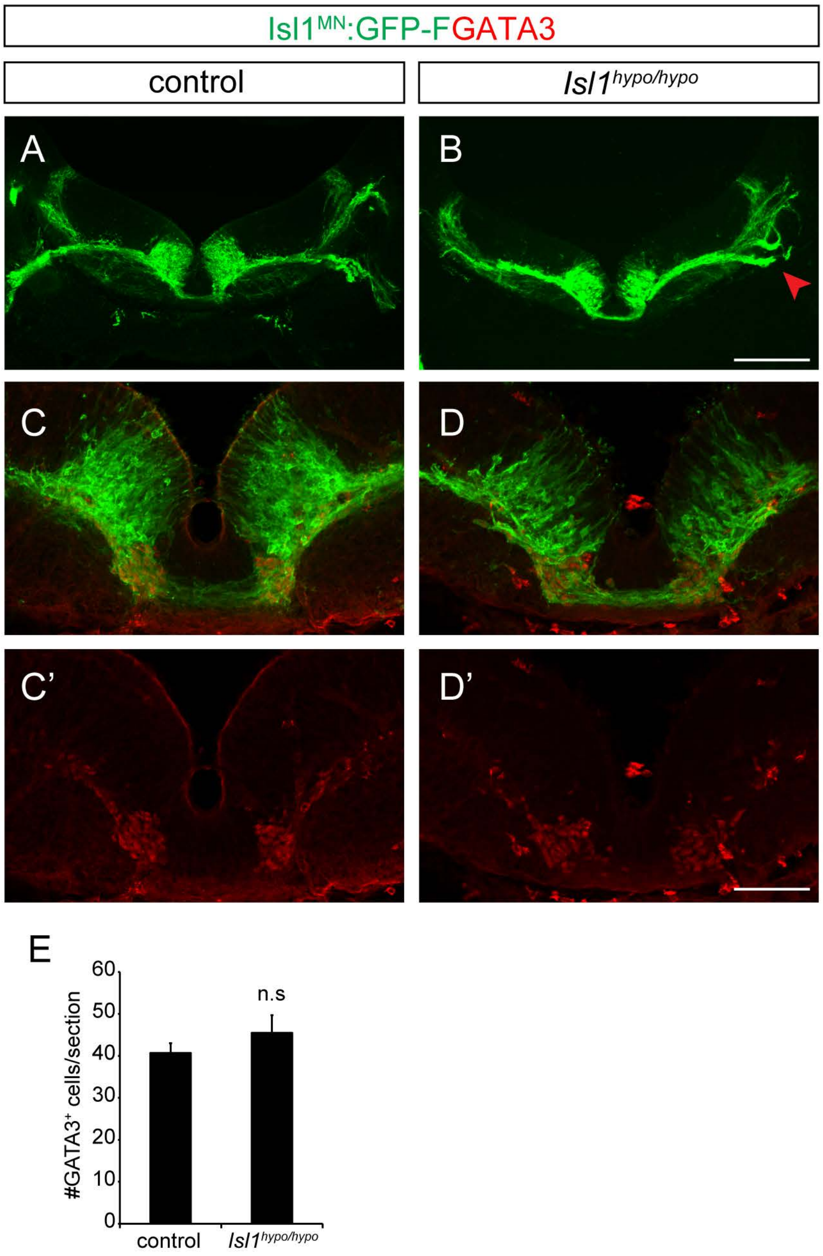
Isl1MN:GFP-F GATA3
control
Isl1hypo/hypo
A
B
C
D
C'
D'
E
60
50
40
30
20
10
0
n.s
#GATA3+ cells/section
control
Isl1hypo/hypo

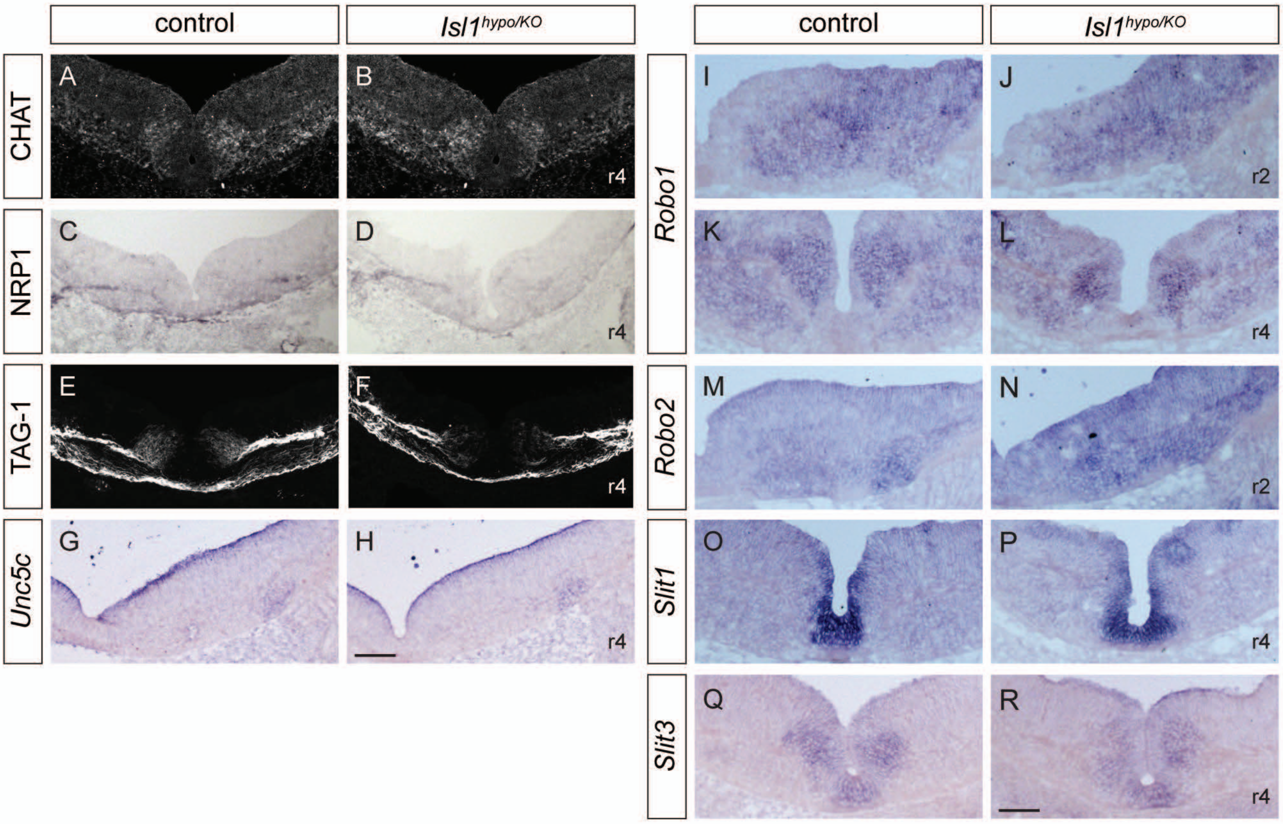
control
Isl1hypo/KO
CHAT
NRP1
TAG-1
Unc5c
Robo1
Robo2
Slit1
Slit3
A
B
C
D
E
F
G
H
I
J
K
L
M
N
O
P
Q
R
r4
r2

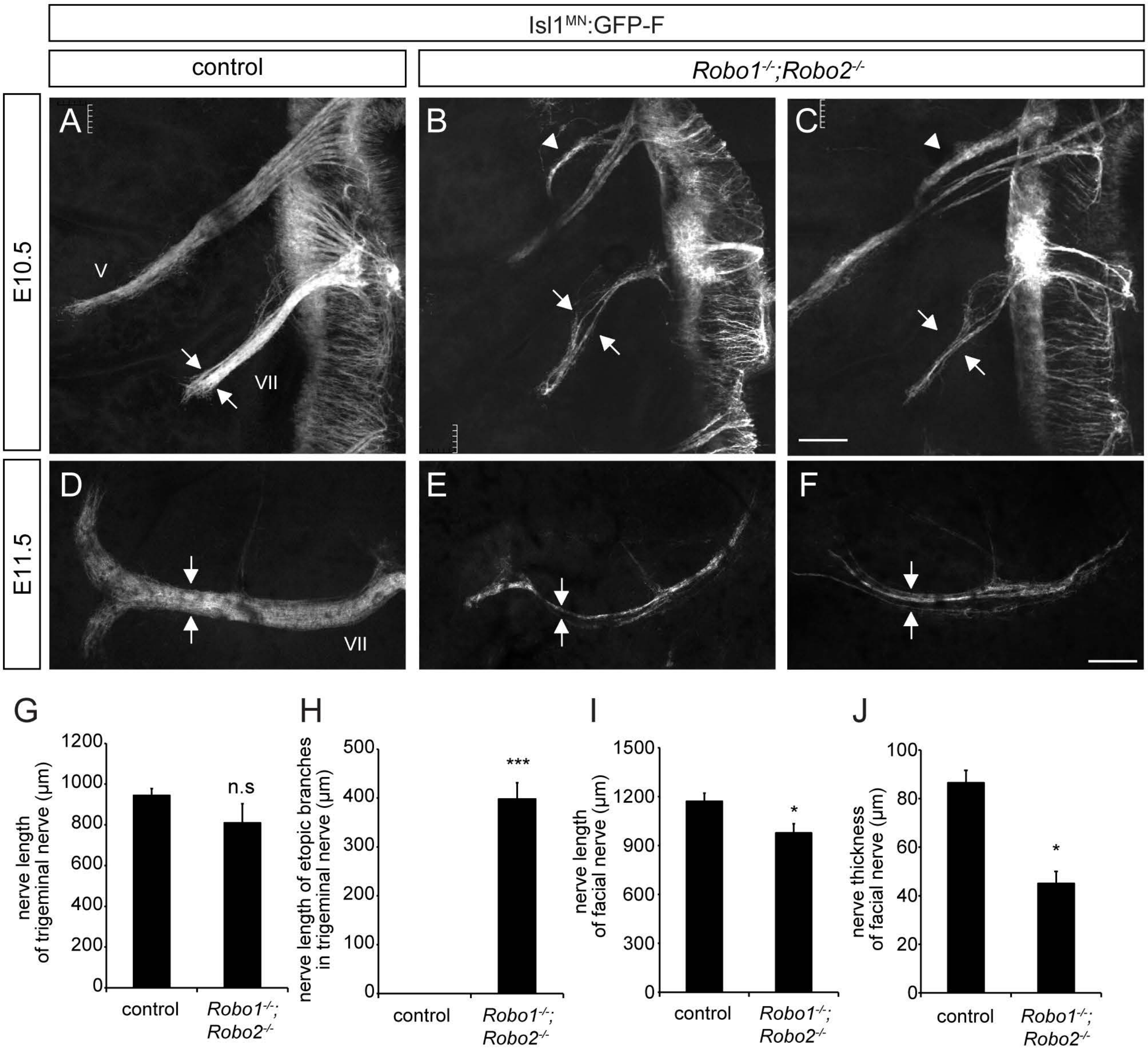
Isl1MN:GFP-F
control
Robo1-/-;Robo2-/-
E10.5
E11.5
A
B
C
D
E
F
V
VII
VII
G
nerve length
of trigeminal nerve (µm)
1200
1000
800
600
400
200
0
n.s
control
Robo1-/-; Robo2-/-
H
nerve length of etopic branches
in trigeminal nerve (µm)
500
400
300
200
100
0
***
control
Robo1-/-; Robo2-/-
I
nerve length
of facial nerve (µm)
1500
1200
900
600
300
0
*
control
Robo1-/-; Robo2-/-
J
nerve thickness
of facial nerve (µm)
100
80
60
40
20
0
*
control
Robo1-/-; Robo2-/-

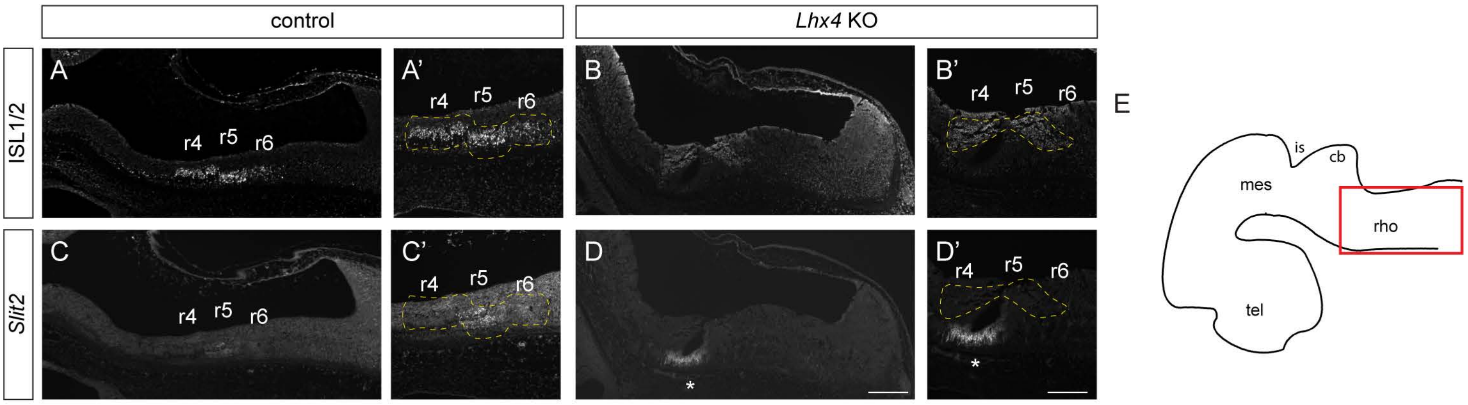
control
Lhx4 KO
ISL1/2
Slit2
A
r4
r5
r6
A'
B
B'
C
C'
D
D'
*
E
is
cb
mes
rho
tel
